# Emerging strategies and novel therapeutic targets in acute myeloid leukemia: current advances and future directions

**DOI:** 10.1186/s40364-025-00839-2

**Published:** 2025-10-14

**Authors:** Linyu Cao, Zhe Wang, Yimin Cui, Yuan Li, Qian Xiang

**Affiliations:** 1 Institute of Clinical Pharmacology, Peking University First Hospital, Beijing, China; 2 Department of Hematology, Peking University First Hospital, Beijing, China

**Keywords:** Acute myeloid leukemia, Immunotherapy, Targeted therapy, Epigenetics

## Abstract

Acute myeloid leukemia (AML) is an aggressive hematologic malignancy characterized by dysregulated differentiation and uncontrolled proliferation of myeloid precursor cells. AML is the second most common type of leukemia after acute lymphoblastic leukemia, yet it has the lower survival rates, with only approximately 30% of adult patients surviving five years post-diagnosis. Standard treatment regimens typically include intensive chemotherapy, advances in allogeneic hematopoietic stem cell transplantation (allo-HSCT) have significantly improved outcomes in the treatment of AML. Advances in molecular profiling technologies have significantly enhanced our understanding of the genetic and epigenetic alterations that drive AML, revealing numerous novel therapeutic targets. Consequently, targeted molecular therapies and epigenetic treatments are becoming increasingly important. Moreover, immunotherapy represents a promising therapeutic strategy that has demonstrated considerable potential in the context of AML. This review summarizes new strategies and emerging therapeutic targets in AML, with a particular focus on recent advancements in immunotherapy. It also explores the feasibility of integrating these therapeutic approaches into current treatment paradigms and their potential impact on future clinical practices.

## Introduction

Acute myeloid leukemia (AML) is a heterogeneous and invasive hematologic malignancy originating from malignant hematopoietic precursor cells in the bone marrow [1–3]. AML tumors contain primitive and differentiated cells. Primitive AML cells, commonly referred to as leukemia stem cells (LSCs), sustain the disease and display stem cell properties such as self-renewal, quiescence, and therapy resistance [4]. Differentiated AML cells lack self-renewal capacity but can impact tumor biology through pathological effects on the tumor microenvironment or hematopoietic function.

AML is influenced by a combination of environmental exposures and genetic predispositions. Environmental risk factors include exposure to chemicals, ionizing radiation, tobacco use, and prior chemotherapy or radiation treatments [5], On the genetic side, certain inherited or acquired genetic abnormalities, such as Down syndrome, Fanconi anemia, Bloom syndrome, and mutations in key genes, including *CEBPA*, *DDX41*, and *RUNX1*, are associated with AML. Additionally, individuals with precursor hematologic conditions, including myelodysplastic syndromes (MDS), myeloproliferative neoplasms (MPN), paroxysmal nocturnal hemoglobinuria (PNH), and aplastic anemia, may be at increased risk for progression to AML [6].

AML is the predominant form of acute leukemia affecting adults, with the median age of diagnosis being approximately 68 years. The prognosis is moderate, with an estimated 5-year survival rate of approximately 30% [7, 8]. However, patients aged 60 years and above have a particularly poor prognosis, with a 5-year overall survival (OS) rate of less than 10% [9, 10].

For the past five decades, the standard treatment for AML has been the “7 + 3” chemotherapy regimen, which combines cytarabine with anthracycline antibiotics, such as daunorubicin or idarubicin. Nevertheless, relapse, treatment refractorines, and emergence of novel resistance variants remain major impediments to successful therapy. AML is characterized by profound cytogenomic heterogeneity, diverse treatment susceptibility, and variable clinical outcomes. The elucidation of its molecular architecture, accelerated by next-generation sequencing, has revealed a typical mutational burden of approximately 13 alterations per genome, with around 5 recurrent driver mutations involving genes such as *FLT3*,* NPM1*,* DNMT3A*,* IDH1*,* IDH2*,* TET2*,* RUNX1*,* TP53*, and *NRAS* [11]. These discoveries have progressively incorporated molecular profiling into prognostic systems and therapeutic guidelines. Since 2017, the therapeutic landscape of AML has been reshaped by the approval of multiple targeted agents directed against molecularly defined subtypes, including FLT3 inhibitors, IDH1 inhibitor, IDH2 inhibitors, and the combination of hypomethylating agents (HMAs) with Bcl-2 inhibitor venetoclax (Ven) have provided new avenues for broader patient cohorts. The development of immunotherapy for AML encompasses a diverse and rapidly evolving spectrum of therapeutic approaches. These range from antibodies targeting AML cells, immune checkpoint inhibitors—which aim to reactivate exhausted T cells-to antigen—specific vaccines designed to elicit targeted immune responses, and advanced cellular therapies such as engineered chimeric antigen receptor (CAR)-T cells and natural killer (NK) cell-based treatments. Although significant progress has been made in preclinical and clinical settings, substantial challenges remain, including the heterogeneity of AML, tumor microenvironment-mediated immunosuppression, and antigen escape. Current research is vigorously focused on refining target selection, developing mechanisms to counteract immune evasion, and designing rational combination strategies that integrate immunotherapies with conventional chemotherapy, targeted agents, or other novel therapeutics. The ultimate goal is to enhance the durability and specificity of anti-leukemic responses, thereby improving long-term survival outcomes for AML patients.

This review outlines the evolving landscape of novel strategies and emerging therapeutic targets in AML, with a dedicated focus on immunotherapeutic advancements. A critical discussion on the integration of these modalities into current paradigms and their potential to catalyze a paradigm shift in clinical management is also provided.

## Standard therapy for AML and their limitations

### Chemotherapy

Anthracycline and cytarabine chemotherapy has been the backbone of induction chemotherapy for AML for more than 50 years [12]. The historical and current standard induction therapy for patients with AML who are suitable for intensive chemotherapy consists of cytarabine (administered at 100–200 mg/m^2^ per day by continuous intravenous (IV) infusion for days 1–7) and an anthracycline (either daunorubicin or idarubicin on days 1–3). This regimen, often referred to as “7 + 3”, was first approved after the landmark study published in 1973 by James Holland’s group at Roswell Park Memorial Institute. This breakthrough dramatically changed the prognosis for patients, demonstrating impressive complete remission (CR) rates of 63% [12]. Despite this development and current CR rates with “7 + 3” currently being closer to 75%, the long-term cure rate with this regimen remains poor, at approximately 30% in younger, fit-for-intensive-chemotherapy patients [9, 13]. In patients older than 60 years and fit for intensive chemotherapy, the median OS (mOS) is approximately 9 months, and the 5-year OS is 10% or less [14]. While 50% to 85% of adult patients may initially achieve CR with induction chemotherapy, primary resistance occurs in approximately 20% of patients. Moreover, the relapse rate is particularly high, with over 50% of younger patients and up to 90% of elderly patients experiencing relapse [15]. Refractory/relapsed (R/R) AML is characterized by heterogeneity and poor prognosis, with a 5-year survival rate of approximately 10% [2, 16]. The incidence of R/R AML is increasing, presenting a significant clinical challenge. Addressing the complexities of relapsed and refractory AML remains a critical focus in oncology research and treatment development. Multi-drug resistance (MDR) presents a major challenge in the treatment of AML [10, 17], mechanisms include intrinsic dormancy mechanisms of LSCs, overexpression of ABC transporters, defects in apoptotic signaling, metabolic reprogramming and epigenetic variations [18].

Historically, options were limited for patients deemed less fit for intensive therapy. Advances in our understanding of leukemia biology and the development of targeted agents against leukemia driver mutations have revolutionized therapy for subsets of patients [8]. The molecular landscape of AML has evolved in the past two decades, and newer induction therapy strategies are being developed to reflect this shift. Targeted therapies are now available for select mutations commonly found in AML (*FLT-3*, *IDH1/2*, *NPM1*, and *KMT2A*), and ongoing trials are evaluating the use of these agents in combination with standard induction strategies. In addition, the development of more effective and less toxic induction regimens with a backbone of Ven and HMAs is a real advance in therapy for older or less fit patients with AML [15].

The safety and efficacy of escalating doses of Ven in combination with “7 + 3” has been demonstrated by multiple groups, with single-arm, short-term results. In a phase II trial involving 33 adults aged 18–60 [19], patients received daunorubicin (60 mg/m² days 1–3), cytarabine (100 mg/m² days 1–7), and Ven (100 mg once daily on day 4, 200 mg once daily on day 5, and 400 mg once daily on days 6–11). The composite CR rate after one cycle was 91%. Outcomes for measurable residual disease (MRD), event-free survival (EFS), OS, and adverse events (AEs) are still pending. A phase Ib trial further demonstrated that adding Ven (400 mg daily for 8, 11 or 14 days) to “7 + 3” induction was safe and highly effective, inducing MRD-negative remissions [20]. The CR rate was 80%, rising to 85.3% in an expansion cohort evaluating dose and duration optimization. Among those achieving CR, 86% were MRD-negative [20]. mOS in all patients was not reached at a follow-up of 9.6 months. When mOS was evaluated by European Leukemia Net (ELN) risk, mOS was 18.5 (9.2, not reached) months. The 30- and 60-day mortality rate was 0%.

These small, single-arm trials demonstrate the safety and CR rates of adding Ven to “7 + 3”, achieving CR rates superior to historical controls. Long-term data on rates of stem cell transplant, recurrence free survival (RFS) rate, and OS will take time to mature. Ongoing, randomized studies through the National Cancer Institute MyeloMATCH program are designed to evaluate the efficacy and toxicity of adding Ven to “7 + 3” and to CPX-351 [21].

### Allo-HSCT

Allogeneic hematopoietic stem cell transplantation (allo-HSCT) is a therapeutic approach for AML that leverages the graft-versus-leukemia (GvL) effect, which is orchestrated by the actions of NK cells and T cells. This treatment modality is efficacious for the vast majority of AML subtypes, with the notable exception of acute promyelocytic leukemia. When considering allo-HSCT, it is imperative to meticulously weigh the safety and therapeutic benefits of the procedure [22].

However, treatment failure in allo-HSCT can arise from disease relapse, graft-versus-host disease (GvHD) [23], delayed immune reconstitution, infections, and conditioning regimen toxicity [2, 24]. According to data from the Center for International Blood and Marrow Transplant Research (CIBMTR), the 1-year survival rate for 1,788 AML patients who experienced relapse post-transplantation was only 23%. The median time to relapse following allo-HSCT is approximately 7 months, with 43% of patients relapsing within the first 6 months post-transplant [25].

Relapse occurs due to immune evasion, where leukemic cells downregulate human leukocyte antigen (HLA) molecules or overexpress immune checkpoint proteins such as PD-L1, reducing donor T-cell activity. Additionally, preconditioning regimens trigger danger signals (DAMPs such as HMGB1 and ATP), amplifying inflammation and disrupting immune homeostasis. Insufficient regulatory T (Treg) cell function and excessive effector T-cell activation further compromise immune control, enabling leukemic cell survival and proliferation [26].

Currently, the main treatment methods for recurrence after transplantation include salvage chemotherapy and secondary transplantation [27], but the recurrence rate and long-term survival rate are low. Alternatively, donor lymphocyte infusion (DLI) can be chosen [28]. DLI technology is an adoptive cellular immunotherapy that involves infusing peripheral blood lymphocytes from a healthy donor into the patient’s body to induce GvL effects, thereby eliminating residual white blood cells in the patient’s body for the treatment of relapse. A retrospective analysis of 399 AML patients who experienced their first hematological relapse after receiving allo-HSCT revealed that DLI increased the 2-year survival rate to 21%±3% compared with 9%±2% for patients who did not receive DLI [29]. The combination of Aza with DLI is a recognized treatment for the relapse of myeloid malignancies after allo-HSCT. Clinical trials, such as NCT02472691, have confirmed its efficacy. Lenalidomide (Lena), known for its immunomodulatory and antileukemic properties, when used in conjunction with Aza and DLI, can improve patient outcomes [30]. The rapid advancement of cellular immunotherapies and targeted drug therapies offers new avenues for improving outcomes in relapsed patients. Integrating these novel approaches with allo-HSCT to optimize long-term survival in AML patients represents a critical area of ongoing research. The development of strategies that enhance immune modulation while minimizing relapse and treatment-associated toxicity will be pivotal in improving the therapeutic landscape for this high-risk population [24, 26, 31].

Following allo-HSCT, GvHD remains a major cause of non-relapse mortality (NRM). This multi-organ syndrome occurs when donor T cells recognize recipient antigens via host antigen-presenting cells (APCs), triggering immune-mediated damage to the skin, gastrointestinal tract, liver, and lungs. Despite its critical role in GvL effects, achieving the balance between GvHD control and preserved GvL remains challenging. Effective prevention strategies are essential for transplant success and long-term patient survival [26].

## Current advanced therapies

### Small molecule therapies

The emergence of molecular studies has revolutionized the diagnostic and therapeutic landscape of AML. AML treatment is shifting from standardized chemotherapy to personalized approaches guided by molecular profiles and targeted therapies. Patients who achieve remission following induction-intensive therapy require consolidation treatment to prevent relapse. This may include targeted therapy; FDA-approved molecular targeted drugs, including FLT3, IDH1, and IDH2 inhibitors; combinations of HMAs with low-dose chemotherapy; Bcl-2 inhibitors; hedgehog inhibitor; potentially, drugs such as mitoxantrone or etoposide highlighting the precision of AML management (Table 1). A notable shift has occurred in the management of older adults unfit for intensive chemotherapy. Previously limited to HMA monotherapy, if treated at all, this population now experiences improved survival with the approval of Ven-based combinations, which have considerably expanded treatment eligibility and clinical benefit. Consequently, molecularly targeted agents are increasingly integrated into both frontline induction and salvage therapies, marking a transition toward precision medicine in AML.

**Table 1:** Approved drugs for AML

Drug (originator)	Description	Targeted Mutations	Route
Midostaurin (Rydapt)	FLT3 kinase inhibitor	FLT3 mutations	Oral use
Gilteritinib (Xospata)	FLT3 kinase inhibitor	Relapsed or refractory FLT3-positive AML	Oral use
Ivosidenib (Tibsovo), Olutasidenib (Rezlidhia)	IDH1 inhibitor	IDH1 mutations	Oral use
Enasidenib (Idhifa)	IDH2 inhibitor	IDH2 mutations	Oral use
Gemtuzumab ozogamicin (Mylotarg)	Anti-CD33 ADC	CD33-positive AML	intravenous injection
Venetoclax (Venclexta)	BCL-2 inhibitor	Various AML subtypes	Oral use
Quizartinib (Vanflyta)	FLT3 kinase inhibitor	FLT3-ITD-positive AML	Oral use
Glasdegib (Daurismo)	Hedgehog pathway inhibitor	Combination therapy for AML	Oral use
Azacitidine/Decitabine	Hypomethylating agents	Combination with targeted therapies	subcutaneous injection
CPX-351 (Vyxeos)	Liposomal cytarabine and daunorubicin	Therapy-related AML, AML with myelodysplasia-related changes	intravenous injection
Revumenib	Menin inhibitors	R/R acute leukemia harboring KMT2A (MLL) rearrangements or NPM1 mutations	Oral use

**Table 2:** Novel immunotherapy under development

Target	Durg	Construct design	Phase	NCT Number	Ref
CD123	Tagraxofusp	recombinant fusion protein	Ⅰb	NCT03113643	[110]
	MP0533	DARPin	Ⅰ	NCT05673057	[112]
	IMGN632	ADC	I/II	NCT03386513	[125]
	AZD9829	ADC	I/II	NCT06179511	[126]
CD30	Brentuximab vedotin	ADC	I	NCT01830777	[127]
CD3-CD33	AMG330	BiTE	I	NCT04478695; NCT02520427	[151]
	AMV564	TandAb	I	NCT03144245; NCT04128423	[154]
	JNJ-67571244	BsAb	I	NCT03915379	[160]
CD3-CLEC12A	MCLA-117	Biclonics^®^	I	NCT03038230	[156]
CD3-CD123	APVO436	BiTE	Ib/II	NCT03647800, NCT04973618, NCT06634394	[157, 158]
	MGD006	DART	I/II	NCT02152956	[164]
PD-1	nivolumab	Antibody	I/II	NCT03825367	[128]
CTLA-4	ipilimumab	Antibody	Ib	NCT01822509	[131]
TIM-3	MBG453	Antibody	II	NCT04623216	[132]
CD47	magrolimab	Monoclonal antibody	III	ENHANCE-2 ENHANCE-3	[135, 136]
CD70	cusatuzumab	Monoclonal antibody	I/II	NCT03030612	[137]
CD45	I-131-Apamistamab	Monoclonal antibody	III	NCT02665065; NCT00589316	[138–140]
CD33-CD16-IL15	GTB-3550	TriKE	I	NCT03214666	[165]
CD33	PRGN-3006	CAR-T	I/II	NCT03927261	[175]
WT1	galinpepimut-S	vaccine	III	NCT04229979	[191]
CD123-NKp46-CD16	SAR443579	NKCE	I/II	NCT05086315	[181]
Clever-1	bexmarilimab	monoclonal antibody	I	NCT05428969	[34]

Recent studies emphasize the feasibility of personalized medicine in AML, where genomic insights guide therapeutic choices, marking an advancement toward precision oncology medicine in the AML field [3]. Menin protein (encoded by the *MEN1* gene) is a scaffold protein that plays a central role in epigenetic regulation. It forms complexes with MLL/KMT2A proteins or mutant *NPM1*, and by aberrantly maintaining the expression of *HOX* gene clusters (such as *HOXA9* and *MEIS1*), it drives the occurrence of leukemia. Revumenib, the world’s first menin inhibitor, received approval from the FDA in November 2024. Compared with those of previous therapies, the remarkable clinical benefits and strong efficacy of Revumenib represent major advancements, and Revumenib may become an important new treatment option for these patients [32].

CK1α is a serine/threonine kinase that negatively regulates β-catenin and p53 signaling. Its inhibition activates p53 and triggers apoptosis, as evidenced by the fact that genetic inactivation of CK1α induces rapid apoptosis in both normal and leukemic hematopoietic cells—a response suppressed by p53 pathway inhibitors. Preclinical studies further indicate that AML cells are more sensitive to pharmacological CK1α inhibition than normal hematopoietic cells [33, 34]. Although Lena promotes CK1α degradation via ubiquitination and activates p53-mediated apoptosis, it has failed to demonstrate sustained efficacy in AML clinical trials, either as monotherapy or in combination. This suggests that CK1α degradation alone may be insufficient to kill AML cells, potentially due to compensatory activation of oncogenic Wnt/β-catenin signaling and MDM2, which antagonizes p53 [35]. Thus, optimizing CK1α-targeted medicinal chemistry holds promise for clarifying its pharmacological profile and unlocking its broad anticancer potential, particularly in AML.

BTX A51, a first-class oral small molecule inhibitor of CK1α and cyclin-dependent kinases (CDKs) 7 and 9, induces the apoptosis of leukemic cells by activating p53 and inhibiting the expression of Mcl1. In a phase I clinical trial of BTX A51 in patients with R/R AML and MDS [36], 3 patients (10%) experienced complete remission with incomplete count recovery (CRi). All 3 responding patients had *RUNX1* mutations, and the CR/CRi rate for *RUNX1*-mutated patients receiving BTX A51 at efficacious doses (11 mg or higher) was 30%. Although the overall efficacy was modest, this study lays the groundwork for future studies with improved patient selection and combination approaches.

### Targeting epigenetics for AML treatment

Epigenetics encompasses heritable and stable changes in gene expression caused by external chromosomal modifications without altering the underlying DNA sequence. Epigenetic modifications, which are established during early development and maintained through successive cell divisions, play a critical role in regulating gene expression. In recent years, with a deeper understanding of the molecular biology of AML, the role of epigenetic regulation in AML has increasingly gained attention. Epigenetic alterations, such as DNA methylation or histone modifications, may play a significant role in the initiation and progression of AML. Numerous studies have shown that in AML patients, 70% of recurrent mutations occur in factors that regulate gene expression, such as epigenetic proteins, transcription factors, and components of the splicing machinery [11, 37–39]. Furthermore, various epigenetic therapies, including DNA demethylating agents, have been approved by global regulatory agencies or have entered the early stages of clinical trials.

#### DNA methyltransferases (DNMTs)

DNMTs are responsible for catalyzing the methylation of DNA, with DNMT1 maintaining existing methylation patterns, whereas DNMT3A and DNMT3B primarily mediate de novo methylation at new sites. In AML, mutations in DNMT3A are very common and are associated with poor prognosis [40]. Inhibiting DNMTs can lead to the re-expression of tumor suppressor genes; therefore, DNMT inhibitors such as decitabine (Dec) and Aza have been approved for the treatment of AML.

#### Histone deacetylases (HDACs)

HDACs remove acetyl groups from histones, typically resulting in a more condensed chromatin structure that represses gene transcription [41]. HDAC inhibitors can relax chromatin, promoting the expression of genes, particularly tumor suppressor genes, whose expression is abnormally silenced [42–44]. However, these inhibitors do not seem to be successful as monotherapies but instead achieve results when used in conjunction with other medications [45]. For example, drugs such as vorinostat and panobinostat have shown efficacy in clinical trials [46, 47]. However, the optimal method of combined treatment still needs to be explored. S1203 was a randomized multicenter trial (NCT01802333) for previously untreated patients aged 18–60 years with AML that compared daunorubicin and cytarabine (DA), idarubicin with higher-dose cytarabine (IA) and IA with vorinostat. The primary endpoint was EFS. The use of higher-dose cytarabine during induction therapy in younger patients with AML, with or without vorinostat, does not result in improved outcomes [48].

#### Lysine acetyltransferase 6A (KAT6A)

Lysine acetyltransferase 6A (KAT6A, also known as MOZ, MYST3) is a member of the MYST family [49]. KAT6A can act as an acetyltransferase to promote H3K9 acetylation. H3K9ac is recognized by ENL, thereby promoting the transcription of related genes and inhibiting the differentiation process of AML. Inhibiting the activity of KAT6A can serve as a potential therapeutic drug for AML [50].

NUP98 fusion oncoproteins (FOs) are hallmarks of childhood AML. NUP98 FOs drive leukemogenesis through phase-separated condensate formation and maintenance of an active chromatin landscape at stem cell-associated genes in cooperation with epigenetic regulators. KAT6A and KAT7 are associated with NUP98 FOs and jointly drive the occurrence of leukemia. Inhibiting their histone acetyltransferase activity is an effective therapeutic strategy for NUP98 rearrangement leukemia (including leukemia resistant to Menin inhibitors). Moreover, combined inhibition of KAT6A/7 and Menin has a synergistic effect, supporting clinical translation to improve the efficacy of leukemia driven by NUP98 FOs [51].

#### Enhancer of zeste homolog 2 (EZH2)

EZH2 is a component of the PRC2 complex, which silences gene expression by adding trimethylation to histone H3 lysine 27 (H3K27me3) [52]. Activating mutations in EZH2 are relatively common in certain types of AML [53, 54]. Small-molecule inhibitors targeting EZH2, such as Tazemetostat, are under clinical investigation to evaluate their efficacy in patients harboring EZH2 mutations [55].

#### DOT1L (disruptor of telomeric silencing 1-like)

Disruptor of telomeric silencing 1-like (DOT1L) is a key hub in histone lysine methyltransferase and an attractive therapeutic target for treating hematological malignancies, including AML [56]. DOT1L is the only known methyltransferase that specifically adds methylation to histone H3 lysine 79 (H3K79). H3K79 methylation is associated with various hematologic malignancies, including AML with MLL rearrangements [57]. EPZ-5676, a selective DOT1L inhibitor, has been demonstrated to be effective against this type of AML in preclinical studies [58].

#### TET (ten-eleven translocation)family proteins

TET family proteins oxidize 5-methylcytosine (5mC) to 5-hydroxymethylcytosine (5hmC), which can be further converted to unmethylated cytosine, thereby facilitating active DNA demethylation [59, 60]. Mutations in *TET2* are also common in AML and may impact the function of hematopoietic stem cells, contributing to leukemia development [61, 62]. Although direct drug development targeting TET proteins is still in its early stages, enhancing or restoring TET function represents a promising future research direction.

#### SUMOylation (small ubiquitin-related modifier)

The small ubiquitin-related modifier (SUMOylation) system is a reversible posttranslational modification (PTM) mechanism that alters target protein interaction surfaces through covalent binding to lysine residues, thereby influencing protein structure and function. Emerging evidence suggests that SUMOylation plays a significant role in AML pathogenesis and treatment response and represents a promising therapeutic target for advanced disease patients [63].

#### Other epigenetic regulators

In addition to the targets mentioned above, many other epigenetic regulators, such as BRD4 (bromodomain-containing protein 4), recognize and bind to acetylated histones, participating in the regulation of gene expression. Small-molecule inhibitors targeting BRD4, such as JQ1, have also shown potential in AML research [64]. The BRD4-targeted PROTAC molecule dBET57 promotes translational readthrough by degrading GSPT1 [65]. dBET57 exhibited significant antiproliferative activity against AML and non-Hodgkin lymphoma (NHL) cells across a diverse panel of tumor cell lines.

### Immunotherapy for AML

Immunotherapy has revolutionized the traditional model of cancer treatment by mobilizing different immune cells in the body to monitor, recognize, and eliminate cancer cells. Changes in immune responses play crucial roles in the pathogenesis of AML, suggesting new options for immunotherapy. AML cells alter the immune microenvironment through various mechanisms, including the upregulation of immune checkpoints and the downregulation of human leukocyte antigen (HLA) classes I and II [66], thereby allowing them to evade immune surveillance. Dysfunction of type 1 innate lymphoid cells (ILC1s) has also been observed in AML [67]. The only immunotherapy drug currently approved for the treatment of AML is gemtuzumab ozogamicin (GO), but its efficacy in R/R AML is limited. In recent years, emerging drugs and treatment strategies, including fusion proteins, monoclonal antibodies, bispecific antibodies, trispecific antibodies, antibody‒drug conjugates (ADCs), immune checkpoint inhibitors, CAR-T cells and NK cell-based treatments have provided more possibilities for the treatment of AML.

#### Targeting key biomarkers of relapse

**Fig. 1:**
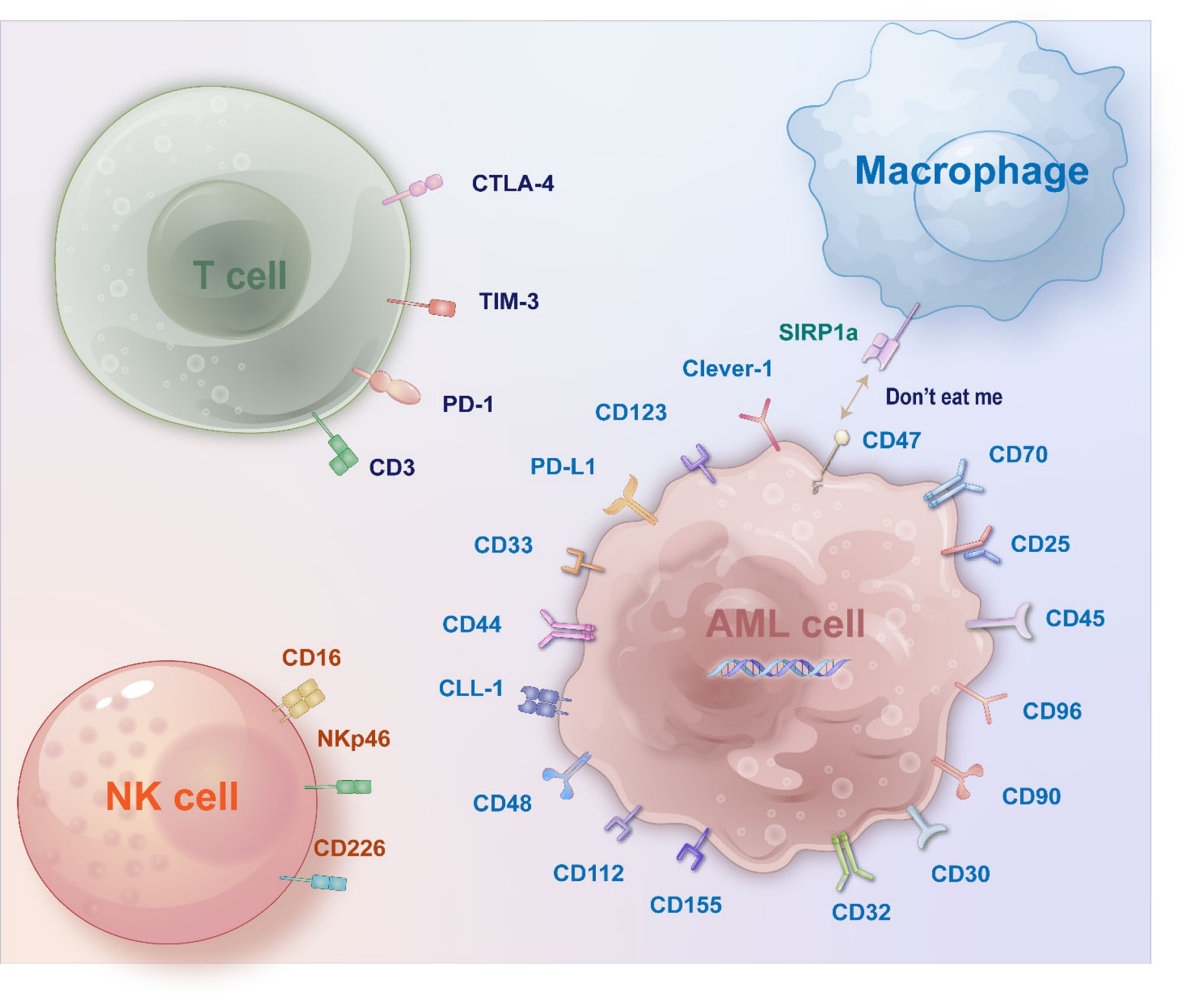
Potential Immunotherapy targets for AML. Immunotherapy for AML is moving toward a more personalized, precise, and integrative approach. With the emergence of new technologies and the advancement of clinical trials, immunotherapy has become a core component of AML treatment, significantly improving patient survival and quality of life

At present, the target antigens specifically expressed by AML cells still need to be determined. Identifying AML-specific antigens can effectively improve the efficacy of immune-targeted therapy and prevent nontargeted leukemia toxicity. Lineage-specific antigens limited to the myeloid lineage not only play a role in the diagnosis of AML but also serve as important targets for the development of new therapies [68]. LSCs can self-renew indefinitely and produce many daughter cells with specific phenotypes of CD33, CD123, CD19, CLL-1, CD44, CD70, CD96, CD90, CD32, and CD25, which are among the most important causes of leukemia recurrence [4] (Fig. 1). Treatment strategies targeting these antigens can more precisely target AML cells, reducing damage to normal cells and improving therapeutic outcomes.

CD33 is a 67 kDa glycosylated transmembrane protein and belongs to the sialic acid-binding immunoglobulin-like lectin (Siglec) family. CD33 is activated upon cross-linking or ligand binding, mediating inhibitory signals that regulate intracellular calcium mobilization, cell adhesion, leukemic cell apoptosis, myeloid cell maturation, and cytokine production [69]. CD33 is expressed on myeloid cells and is particularly highly expressed on the surface of AML cells (85% to 90%), whereas CD33 is not expressed on the surface of hematopoietic stem cells (HSCs), mature granulocytes or other tissues, making it highly valuable for the treatment of AML [70]. Myeloid-derived suppressor cells (MDSCs) contribute to an immunosuppressive tumor environment and are a barrier to immune therapy. CD33 signaling in immature myeloid cells promotes the expansion of MDSCs and the production of immunosuppressive factors [71].

CD123 is a transmembrane glycoprotein that can specifically bind to IL-3 with low affinity as a monomer. When it forms a heterodimer with the β chain of IL-3R (CD131), it can form a high-affinity IL-3 receptor complex and activate downstream signaling of the intracellular receptor cascade through the cell membrane, thereby activating the JAK/STAT, RAS-MAPK, and PI3K pathways. Moreover, CD123 is widely overexpressed in various hematological malignancies [72], including AML, B-cell acute lymphoblastic leukemia(AIL), hairy cell leukemia, Hodgkin’s lymphoma (HL), and myeloid dendritic tumors (BPDCNs), mainly in CD34^+^/CD38^−^ AML cells [73]; more importantly, CD123 is expressed in both LSCs and more differentiated leukemic blasts. Although CD123 is also expressed in normal hematopoietic stem cells, its expression level is lower than that in LSCs; another study has shown that the overexpression of CD123 suggests a poor prognosis in patients [74]. For these reasons, CD123 has become an attractive new therapeutic target for hematological malignancies.

The initial response to standard chemotherapy in most t(8;21) AML patients, approximately 35% experience relapse or persistent MRD positivity, conferring a dismal 5-year OS rate of ≤ 20% in this setting, This poor prognosis stems from the limited therapeutic options available for this molecularly defined subgroup [75–77]. Approximately 78.3% of patients with t (8;21) AML express CD19 [78], making it a potential target for chimeric antigen receptor (CAR)-T-cell therapy focused on CD19.

In 2004, Bakker first identified CLL-1 (CLEC12A, MICL, DCAL-2 or CD371) via phage display technology, which is overexpressed in 90% to 95% of newly diagnosed or relapsed AML cases and is rare in normal tissues [79]. Notably, CLL-1 expression on LSCs makes it a highly attractive target for treating AML. Previous studies have shown that targeting CLL-1 can treat AML in preclinical studies [80, 81].

CD44 is a highly glycosylated transmembrane glycoprotein that exists in multiple isoforms. CD44 is selectively and highly expressed in hematopoietic and epithelial tumors, and is considered to be an indication of cancer stem cells for multiple cancers [82]. The isoform 6 of CD44, named CD44v6, is relatively tumors-limited and associated with poor prognosis in AML [83–85]. In AML, CD44v6 was selectively expressed in primary cells of AML but not in HSCs, thus ensuring its safety as a CAR‐T therapeutic antigen [85].

CD70 is a transmembrane protein of the tumor necrosis factor family, with CD27 as its receptor molecule, and is expressed primarily on activated T cells and lymphocytes under normal physiological conditions. The interaction between CD70 and CD27 can promote cell activation. CD70 is a promising antigen to target AML, as it is expressed on most leukemic blasts, whereas little or no expression is detectable in normal bone marrow samples [86]. Compared with CD33 and CD123, the greatest advantage of CD70 is that it is exclusively expressed in AML and LSCs, not in HSCs [87].

#### Targeting key immunoregulation biomarkers

Immune checkpoint inhibitors and immunomodulators play pivotal roles in immunotherapy for hematological malignancies [88, 89]. CTLA-4 (CD152), a receptor shared by T-cell receptors (TCRs) and part of the immunoglobulin superfamily, is expressed on CD4^+^ and CD8^+^ T lymphocytes and inhibits immune regulation [88]. CTLA-4 binds to its ligands, CD80 (B7-1) and CD86 (B7-2), which are found on APCs. This binding inhibits the phosphorylation of the ζ chain associated with TCRs, dampening signals to lymphocytes. Consequently, blocking CTLA-4 activity bolsters the immune system’s capacity to identify and eliminate tumor cells [90].

PD-1 (CD279), a coinhibitory molecule of the Ig superfamily, is expressed on activated T cells, B cells, and myeloid cells. Its binding to PD-L1 (CD274), which is found on the surface of tumor cells and MDSCs, diminishes TCR-mediated signaling. This pathway also governs the development, maintenance, and function of induced T-regulatory cells. T cell exhaustion does not play a major role in AML. Immunotherapeutic strategies targeting autologous T cells thus have particularly good prospects in the setting of AML [91].

TIM-3 is a negative regulatory immune checkpoint present in different types of immune cells. The TIM-3 signaling pathway plays an important role in the regulation of T-cell activity and number, and it can participate in tumor immune suppression and progression by mediating apoptosis. By interacting with the ligand galectin-9, it acts as a negative regulator for these lymphocyte populations, triggering cell death. TIM-3 is expressed on immune cells and LSCs but not on normal HSC [92]; its interaction with galectin-9 promotes the self-renewal of LSCs, making it a promising target for AML.

CD47 is a transmembrane protein, and its interaction with signal regulatory protein alpha (SIRP α) determines the inhibitory regulation of phagocytosis mediated by macrophages. A monoclonal antibody against CD47 blocks this interaction, thereby promoting the killing of tumor cells by macrophages and the cross-priming of tumor-specific cytotoxic T cells, which in turn activates the adaptive immune response [93]. CD47 expression by LSCs inhibits macrophage activity and is an indicator of poor prognosis for patients with AML [94].

#### Targeting LSC-associated antigens

To date, there are relatively few approved immunotherapy methods for treating AML compared with AIL, although many methods have shown promising preliminary results in clinical trials. One possible reason for this difference is the difficulty in identifying targeted antigens on bone marrow cells, which need to be safe and able to exert long-lasting effects. Some leukemia-associated antigens are more highly expressed in AML cells than in healthy tissues. However, these antigens may be found in nonhematopoietic tissues, leading to off-target treatment toxicity. Given the high heterogeneity within the AML population, the challenges in identifying antigens specific to LSCs, and the relatively lower antigen density of LSCs than lymphoid cells, determining the optimal therapeutic target presents a major challenge. This complexity is further compounded by the need to decide among various treatment strategies—such as combination chemotherapy and/or molecular therapy or sequential therapy—and between antibody-based and cell-based therapies. Since these antigens are abnormal proteins encoded by leukemia mutations and are often exclusively expressed in malignant clones, they may be ideal targets. Guy’s research, through the analysis of the surface proteome of 100 genetically diverse primary AML samples combined with single-cell transcriptomics, successfully identified a variety of antigens and markers predominantly expressed by primitive AML cells [95]. Subgroups of AML exhibit distinct expression profiles of antigens and primitive cell markers.

Although leukemia patients can achieve a certain degree of remission after initial treatment, almost all patients experience relapse without consolidation therapy. Therefore, people are increasingly concerned about targeted residual chemotherapy, such as persistent leukemia-initiating cells or leukemia stem cells. Resting leukemia cells are resistant to chemotherapy [96], and drug-resistant leukemia stem cells are enriched in the expression of stemness and immune escape genes [97]. Rutella and Coll [98] studied CD8^+^ exhaustion/aging driven by AML, developed immune effector dysfunction (IED) scores associated with leukemia stem cells and poor treatment response, and may predict resistance to immunotherapy. AML tumor cells have multiple immune deficiencies, including the absence of MHC class I molecules, which prevent CD8^+^ T cells from effectively recognizing and killing tumor cells [99, 100], affecting the efficacy of immunotherapies, including immune checkpoint inhibitors (ICIs), T-cell receptor-modified T cells (TCR-T cells), and T-cell-binding bispecific antibodies (T-BsAbs).

Moreover, AML patients have impaired NK cell activity and function, decreased expression of activated receptors, increased expression of the inhibitory receptor NKG2A, and reduced killing ability [101, 102]. In vitro studies have shown that monocytic leukemia cells can induce the apoptosis of T cells and NK cells by generating ROS [103]. In addition, AML cells can inhibit T-cell proliferation, downregulate the expression levels of costimulatory molecules, and induce apoptosis. In leukemia cells with *PML–RARα* and *AML–ETO* mutations, the expression of CD48, a ligand for NK cell receptors on the cell membrane surface, is downregulated, leading to a decrease in NK cell killing activity [102, 104]. Currently, emerging approaches focus on dual-targeting and split co-stimulation strategies.

CD226, an activating receptor predominantly expressed on NK cells, plays a pivotal role in innate immune surveillance by recognizing and binding to its ligands—CD112 (NECTIN-2) and CD155 (PVR)—which are frequently overexpressed on malignant cells, including AML blasts [105]. This interaction triggers NK cell activation, leading to targeted cytotoxicity against tumor cells [106]. Given its critical function in immune recognition and antitumor response, CD226 has emerged as a promising therapeutic target in AML.

Clever-1 (Common lymphatic endothelial and vascular endothelial receptor-1, also known as Stabilin-1) is a multifunctional large glycoprotein receptor expressed on immunosuppressive macrophages and monocytes, sinusoidal endothelial cells, and lymphatic endothelial cells [107]. Myeloid blasts are the only tumor cells that express *STAB1*, encoding Clever-1. In cell lines and patients with AML, high *STAB1* expression is associated with drug resistance and poor outcomes [108]. Because blocking Clever-1 activity activates antigen presentation and T cells and because immunomodulatory effects have been associated with hypomethylating agent treatment, this combination could lead to favorable clinical activity. Non-clinical findings support the feasibility of CLEVER-1 inhibition in AML/MDS to induce antigen presentating molecule expression and potentially, an anti-leukemic effect together with standard-of-care drugs [109].

## Novel immunotherapy under development

Driven by emerging technologies and progressive clinical trials, immunotherapy has evolved into a cornerstone of AML treatment. As summarized in (Table 2), the development of novel immunotherapeutic agents—including immune checkpoint inhibitors, bispecific T-cell engagers, antibody-drug conjugates, and CAR-T/CAR-NK cell therapies—has expanded the arsenal against AML. These innovations are showing promising efficacy in enhancing antitumor immunity, overcoming resistance, and achieving durable responses. As a result, immunotherapy is not only significantly improving survival rates but also elevating the quality of life for AML patients, marking a transformative shift in the management of this disease.

### Recombinant fusion protein

Tagraxofusp is a recombinant fusion protein consisting of the binding site of IL3 fused with diphtheria toxin, which is specifically designed for the CD123 target [110]. Once internalized by cancer cells, targeted antibody-drug conjugates (TAGs) irreversibly halt protein synthesis, triggering apoptosis in target cells. TAG has received regulatory approval for treating BPDCN. For AML patients who have developed resistance to TAG, re-sensitization can be achieved through Aza treatment, as TAG-exposed cells exhibit increased reliance on the antiapoptotic protein Bcl-2. Encouraging safety and efficacy profiles were observed in a phase Ib clinical trial (NCT03113643) with a combination of TAG, Aza, and Venin high-risk AML patients, including those with *TP53* mutations [111]. These results advocate for the continued clinical exploration of this combined therapeutic approach.

MP0533 is a multispecific CD3-engaging designer ankyrin repeat protein (DARPin) that simultaneously targets three antigens—CD33, CD123, and CD70—on AML cells. This approach aims to enhance T-cell-mediated cytotoxicity through avidity-driven interactions, specifically targeting AML cells that coexpress at least two of these antigens [112]. MP0533 is currently being evaluated in a dose-escalation phase I clinical trial (NCT05673057) in patients with R/R AML.

### Antibody-drug conjugate(ADC)

ADCs kill cancer cells through various methods. The antibody component of ADCs interacts with immune effector cells, inducing antitumor immunity, including CDC (complement-dependent cytotoxicity), ADCC (antibody-dependent cellular cytotoxicity), and ADCP (antibody-dependent cellular phagocytosis) effects. The antibody component retains its activity, thereby disrupting target function, inhibiting downstream signaling pathways, and suppressing tumor growth. On the basis of highly promising preclinical data, several ADCs that target different surface markers and carry different payloads have begun clinical trials for the treatment of AML.

Mylotarg^®^ (gemtuzumab ozogamicin, GO) is an anti-CD33 ADC that delivers calicheamicin—a highly potent DNA-damaging toxin. It holds the distinction of being the first ADC approved worldwide. After the antibody binds to the CD33 antigen, the cytotoxin is internalized and released, which induces double-stranded DNA breaks and cell death. GO received accelerated FDA approval in 2000 for the treatment of AML. Owing to unstable linkers and severe toxic reactions caused by the early release of cell toxins, safety issues were raised, and the market was voluntarily withdrawn in 2010. Subsequently, Pfizer updated the clinical evidence and made corresponding adjustments to the dosing regimen. In 2017, Mylotarg^®^ was approved for the treatment of newly diagnosed myeloid differentiation antigen CD33 positive AML in adults, as well as R/R myeloid differentiation antigen CD33 -positive AML in children and adults aged ≥ 2 years [113]. The addition of GO to intensive chemotherapy has become a mainstay in treating patients with core binding factor AML, patients receiving GO had a 2-year overall survival of 90% compared with 80% in those without GO and a 2-year EFS of 51% vs. 36%. While CR rates in GO vs. non-GO patients were comparable (89% vs. 90%, *P* = 0.81), more GO patients achieved MRD-negative remission (77% vs. 49%, *P* < 0.001), resulting in numerically reduced cumulative incidence of relapse (HR 0.67, 95% CI 0.43–1.02, *P* = 0.06) [114].

The exploratory clinical trials of GO are moving from traditional chemotherapy combinations to novel combination strategies and individualized dosing guided by precise biomarkers. Its future applications will focus more on biomarker-driven patient selection (such as efficacy mining of molecular subgroups) [115–118], rational combination with novel targeted drugs/immunotherapies [119, 120], the value exploration in scenarios of low tumor burden (such as MRD clearance) [121], Continuous optimization of the drug administration plan and dosage [113, 122, 123], feasibility and safety of using GO as a bridging treatment before allo-HSCT or as maintenance therapy after transplantation [124].

IMGN632 is an ADC that targets CD123, comprising a high-affinity anti-CD123 antibody conjugated to a novel IGN-class DNA alkylating agent. A phase I/II clinical trial established the maximum tolerated dose (MTD) and the recommended phase II dose (RP2D) for IMGN632 [125]. This trial assessed the safety, tolerability, pharmacokinetics, pharmacodynamics, immunogenicity, and antileukemic activity of IMGN632 monotherapy in patients with CD123^+^ hematologic malignancies. The results indicated that multiple doses of IMGN632 demonstrated single-agent activity against leukemia with a favorable safety profile. In AML patients with poor risk characteristics who had previously received extensive treatment, the composite CR rate at a RP2D of 0.045 mg/kg reached 17%. These findings support the combination of IMGN632 with various other agents, including Aza and Ven, for the treatment of AML to increase response rates and clinical benefits. Recent triple-combination data with IMGN632, Aza, and Ven have shown broad antileukemic activity. Moreover, an objective response rate (ORR) of 53% was observed in R/R AML patients not treated with Ven, suggesting that this triplet therapy could be evaluated in newly diagnosed AML patients who are not candidates for intensive chemotherapy.

AZD9829 is an ADC composed of an antibody targeting CD123 and a payload of AstraZeneca’s proprietary topoisomerase 1 inhibitor (TOP1i). The main mechanism of action of this drug is to deliver the TOP1i payload to cancer cells expressing CD123, leading to DNA damage and apoptosis. AZD9829 has shown strong killing ability against CD123^+^ AML cell lines in vitro, and the drug has demonstrated significant antitumor activity in 13 xenograft models derived from AML patients with different mutation states and disease stages [126]. In addition, AZD9829 had a persistent effect on reducing leukemia stem cells in the blood and bone marrow on the 28th day after the first administration, which supports its clinical development. Currently, a phase I/II clinical study (NCT06179511) is underway to evaluate the safety, tolerability, pharmacokinetics, and preliminary antitumor activity of AZD9829 alone or in combination therapy for CD123^+^ hematological malignancies.

Brentuximab vedotin (BV), an ADC that targets CD30, has made significant progress in the treatment of hematological malignancies. BV is currently approved for the treatment of recurrent HL and systemic anaplastic large-cell lymphoma (ALCL), as well as post-transplant maintenance therapy for HL. In a phase I trial (NCT01830777), the combination of traditional mitoxantrone, etoposide, and cytarabine reinduction chemotherapy showed good safety and tolerability in CD30^+^ R/R AML patients [127].

### Immune checkpoint inhibitors

To date, immune checkpoint inhibitors have demonstrated efficacy in treating solid tumors and patients with HL; however, they have not achieved the same level of significant success in AML. A variety of these inhibitors are being explored for potential use in AML, both as standalone treatments and in conjunction with established therapeutic regimens. Nonetheless, current evidence indicates that the efficacy of these monoclonal antibodies is somewhat constrained when they are administered in isolation. Nevertheless, there is promising potential for synergistic effects when these compounds are integrated with HMAs.

#### PD-1

The combination of nivolumab and Aza has been shown to improve median survival in adult AML patients. A phase I/II clinical study (NCT03825367) conducted in children with R/R AML evaluated the efficacy of the combination therapy of nivolumab and Aza [128]. The results revealed good tolerability of the combination therapy, and no dose-limiting toxicity was observed. The AEs are mainly hematological, with the most common grade > 3 AE being febrile neutropenia, accompanied by an increase in CD8^+^ T cells, a decrease in CD4^+^/CD8^+^ T cells, and the demethylation of peripheral blood and bone marrow cells. Future research may explore this combination to maintain remission in children at high risk of AML recurrence.

Unlike allo-HSCT in AML through T-cell activity, immune checkpoint inhibitors have limited efficacy, which may be related to the T-cell phenotype and TCR spectrum. The disease-related T-cell subpopulations are highly heterogeneous, and their abundance changes after PD-1 blockade therapy. In patients who respond to treatment or have stable conditions, the TCR library is derived mainly from CD8^+^ T-cell expansion, whereas in treatment-resistant patients, the TCR library contracts. Adaptive T-cell plasticity and genomic changes determine the response of AML patients to PD-1 blockade [129]. However, Targeting PD-1 for reversing T-cell exhaustion and restoring the GvL effect may have logistical advantages over DLI. In a prospective phase Ib clinical trial (NCT03286114), pembrolizumab was administered every 3 weeks to 16 patients with AML (*n* = 12) and MDS (*n* = 4) in relapse after HCT to assess GvHD, clinical response, and survival. In this trial, PD-1 inhibition led to durable remission in one-third of the patients experiencing early relapse after HSCT, 1-year OS was 50.0%, suggesting that this approach may augment the GVL response [130].

#### CLTA-4

A phase Ib study (NCT01822509) assessed the efficacy of ipilimumab in patients with relapsed/refractory AML following allo-HSCT [131]. Among 22 patients, four achieved sustained responses (> 1 year). Notably, 21% of patients experienced immune-mediated toxicity, and 14% developed GvHD.

#### TIM-3

Sabatolimab (MBG453) is a hinge-stable humanized anti-TIM-3 IgG4 ҡ antibody developed by Novartis for the treatment of MDS and AML. The safety and tolerability of sabatolimab + Aza + Ven were comparable at 2 doses (400 and 800 mg) of sabatolimab and to the safety profile of Aza + Ven therapy [132]. Clinical studies of sabatolimab as a treatment for patients with AML and MRD after allo-HSCT (NCT04623216) are ongoing.

### Monoclonal antibodies

Monoclonal antibodies are a major component of cancer therapy. The functional effect of a mAb is related to a cancer antigen profile and the specific ability of the mAb to be internalized, activate Fcγ-receptors on innate immune cells, trigger the activation of complement, or block receptor-mediated oncogenic signaling [133].

Currently, nearly 50 CD47-targeting monoclonal and multispecific antibodies are in clinical development worldwide, positioning CD47 as one of the most promising therapeutic targets in the post-PD-1 era. However, the highly anticipated CD47 monoclonal antibody has not achieved ideal results in the treatment of AML. Magrolimab (Magro) is a humanized immunoglobulin G4 monoclonal antibody that blocks CD47. Its mechanism of action may be to promote the death of immunogenic cancer cells. Aza can upregulate the expression of CD47 and claudynin (an activator of phagocytosis), providing a theoretical basis for the combination of Magro and Aza (Magro/Aza). The in vitro co-culture experiment of *TP53* mutant AML cell lines with human macrophages showed that Magro/Aza could significantly enhance the phagocytic ability of AML cells compared to Aza alone. An Ib-phase study (NCT03248479) demonstrated that in untreated TP53 mutant AML patients (*n* = 72), Magro/Aza was well tolerated and showed significant efficacy [134]. However, in a phase III clinical trial (ENHANCE-2 study) conducted in patients with AML carrying TP53 mutations [135], the results of Magro/Aza failed to replicate. The treatment of AML with TP53 mutations remains a significant challenge. The results of ENHANCE-2 have highlighted the treatment difficulties faced by this refractory population and have provided prospective randomized data on the activity of Ven/Aza. In the phase III ENHANCE-3 clinical trial conducted by magrolimab in AML [136], the data revealed that the combination of magrolimab and Ven with Aza did not result in any survival benefits and increased the risk of death in AML patients. These results highlight the challenges in improving the prognosis of such AML patients. The reason for the increased fatal infections observed in the study remains unclear, especially given that the duration of neutropenia and the overall infection rate were similar in both groups. This suggests that in future first-line treatment trials for elderly/unsuitable-for-intensive-chemotherapy AML patients, standardized infection prevention measures may be needed. Despite the negative results, the ENHANCE-3 study, as the first phase III randomized trial to evaluate anti-CD47 therapy for first-line treatment of AML patients unsuitable for intensive therapy, and the first randomized trial to use Ven combined with Aza as the control group, provided valuable data and benchmarks, offering important references for the future development of anti-CD47 therapy and the design of trials in this field.

Cusatuzumab (ARGX-110) is an anti-CD70 monoclonal antibody. On the basis of preclinical results, a phase I/II trial (NCT03030612) evaluated the efficacy of single-dose cusatuzumab combined with Aza in untreated elderly AML patients [137]. Aza induces CD70 expression on LSCs; therefore, its combination with ARGX-110 is beneficial for in vitro killing. Ten patients (83%) achieved CR/CRi, 4 patients achieved MRD-negative results via flow cytometry, and no dose-limiting toxicity was reported.

I-131-Apamistamab (Iomab-B) is a mouse IgG1κ antibody against CD45 that is radiolabeled with I-131. This antibody is designed to deliver targeted myeloablative radiation to stem cells and leukemic progenitor cells in the bone marrow, serving as a preconditioning therapy before allo-HSCT in patients with active, relapsed, or refractory AML [138]. A phase I/II study (NCT00589316) using Iomab-B in combination with nonmyeloablative conditioning prior to HLA-haploidentical HCT in adults with high-risk R/R AML, ALL or MDS, has shown that the use of Iomab-B as a bridging therapy before transplantation is safe and feasible for patients with R/R AML [139]. In the pivotal phase III SIERRA trial (NCT02665065), an analysis of the first 25% of enrolled patients (*n* = 38) demonstrated that Iomab-B treatment led to a rapid reduction in leukemia burden, laying the groundwork for successful engraftment after allo-HSCT [140].

Blocking Clever-1 with bexmarilimab, a fully humanized IgG4 monoclonal antibody, promotes proinflammatory cytokine secretion, T-cell activation, and antigen presentation in vitro and leads to tumor rejection in vivo. The first-in-human MATINS study (NCT03733990) evaluated bexmarilimab as monotherapy in solid tumors, where single-agent bexmarilimab was well tolerated in 216 patients with advanced solid tumors [141]. The results showed that Clever-1 blockade promoted macrophage reprogramming and T-cell activation, leading to the control of tumor growth. A phase I dose-escalation study (NCT05428969) aimed to select the recommended dose for expansion of bexmarilimab in combination with Aza and reported its safety and preliminary activity in patients with high-risk MDSor R/R AML [34]. The current results showed that bexmarilimab in combination with Aza has a manageable safety profile, which is consistent with that of Aza, and shows promising clinical activity in patients with myeloid malignancies.

### Bispecific antibodies

Bispecific antibodies are formed by connecting two single-chain antibody fragments via the genetic engineering of antibody technology. They can simultaneously bind to tumor cell surface antigens and cytotoxic T lymphocyte (CTL) surface antigens (such as CD3), activate CTLs, and overcome the immune escape mechanism of tumor cells, effectively killing tumor cells. The anti-CD19/CD3 bispecific antibody blinatumomab (Blincyto) has been approved for use in adults with R/R precursor B-cell AIL. Bispecific antibodies also have promising prospects in the treatment of AML.

Bispecific T-cell engager (BiTE) typically consists of two single-chain variable fragments derived from natural antibodies, which simultaneously target tumor antigens and CD3-expressing T cells [142, 143]. Through this approach, BiTE can retarget and activate the T-cell-mediated immune system to combat tumor cells and achieve clinical remission. The molecular weight of BiTEs is 55–60 kDa, which classifies them as small molecules with good permeability, allowing them to reach areas that are difficult for large-molecule antibodies to access and bind to antigens. However, they have a lower affinity and a shorter half-life in the body.

TandAbs are tetravalent antibody molecules with a structure of Fv1-Fv2-Fv2-Fv1, formed by the reverse pairing of two polypeptide chains into a homodimeric molecule [144]. TandAbs have a relative molecular mass of approximately 110 kDa, positioning them between whole-molecule antibodies and BiTEs. TandAbs can bind to two types of antigens, with two binding sites for each antigen. TandAbs can recruit effector cells (T cells or NK cells) and exert a cytotoxic effect on target cells (tumor cells or cancer cells). When TandAbs link T cells to tumor cells, T cells are activated, releasing substances such as perforin, granzymes, and lysosomal enzymes. These substances are transported to the T-cell membrane and secreted into the extracellular matrix. Perforin creates pores in target cells, facilitating the entry of lytic substances, thereby causing lysis of the target cells.

DART (Dual-affinity re-targeting) proteins are heterodimeric antibodies formed by the combination of two polypeptide chains [145]. Their structure is created by linking the VH and VL sequences of one antibody’s variable region with the VL and VH sequences of another antibody’s variable region, respectively. Additionally, cysteines are introduced at the C-termini of both polypeptide chains, and interchain disulfide bonds are formed through these cysteines to increase the stability of the product. DART also activates the immune system of patients receiving treatment by retargeting T cells. However, compared with BiTE, DART molecules carry two specific variable domains in alternating order on two chains. Currently, research on bispecific antibody drugs that target multiple antigens on AML cells is underway.

Biclonics^®^ leverages the attractive characteristics of natural antibodies, such as the common light chain for “unforced, natural pairing with 2 different heavy chains”, the lgG format for efficient manufacturing and predictable in vivo behavior, electrostatic attraction to efficiently drive the formation of bicyclic, Fc modifications for improved functionality [146, 147].

AMG330, developed by Amgen, is a BiTE that targets CD3 and CD33 [148]. It is designed to bridge the CD3 complex on T cells, a key component of the T-cell receptor, with the surface antigen CD33 on tumor cells. This linkage forms a T-cell-BiTE-tumor cell complex, which activates T cells to initiate a tumor-killing response. In vitro studies have demonstrated its ability to eliminate primary AML patient cells across various effector-to-target cell ratios and its capacity to sustain the expansion and activation of T cells [149, 150]. A phase I trial conducted in patients with R/R AML revealed that AMG 330 at a dose of up to 720 µg/day provided early evidence of an acceptable safety profile, drug tolerability, and antileukemic activity [151].

AMV564 is a potent T-cell engager that selectively depletes MDSCs while promoting T-cell activation and proliferation via preferential binding to areas of high CD33 density. By design, AMV564 has reduced clearance and therefore has a longer half-life (t_1/2_) than monovalent, bispecific T-cell engagers. In preclinical investigations using both leukemic cell lines and primary cells from AML patients, AMV564 eliminated myeloid blasts with picomolar potency and broad activity independent of cytogenetic or molecular abnormalities, CD33 expression levels, and disease stage, with no nonspecific activation of T cells [152]. In a phase I clinical trial (NCT03144245) targeting adult patients with R/R AML, no patient died within 30 days of treatment initiation. Bone marrow blast reductions were observed in 17 (49%) of the 35 patients for whom efficacy was evaluable. Objective responses have been observed, including 1 CR during cycle 1 at the 200 mcg/day assigned dose, 1 CRi during cycle 2 at the 150 mcg/day assigned dose, and 1 partial response (PR) during cycle 1 at the 100 mcg/day assigned dose [153]. Moreover, in a phase I clinical trial conducted in solid tumors (NCT04128423), AMV564 depleted MDSC populations and altered T-cell profiles, which was consistent with the activation of cytotoxic T cells and a Th1 response [154].

MCLA117 is an improved full-length human bispecific IgG that binds with high affinity to CLEC12A, is expressed on AML blasts and LSCs, and has a relatively lower affinity for CD3 expressed on T cells. Preclinical data show that it can effectively activate and expand patient T cells and effectively kill patient AML cells [155]. In a phase I multinational study of MCLA-117 (NCT03038230) in patients with AML [156], MCLA-117 was shown to be safe and well tolerated with manageable CRS events. Following a ramp-up dosing scheme, clinical activity was observed with a ≥ 50% blast reduction in BM, including 1 patient who achieved a morphological leukemia-free state.

APVO436 is an innovative bispecific monoclonal antibody developed by Aptevo Therapeutics that can simultaneously target CD123 on the surface of tumor cells and CD3 on the surface of T lymphocytes, thereby redirecting the host immune system’s T cells to the patient’s tumor cells to rapidly and completely destroy tumor cells that express CD123 on their surface. APVO436 targets CD123 on AML cells and redirects CD3^+^ T cells to the vicinity of the target leukemia cells. APVO436, after modification, can remain in the bloodstream for a sufficient amount of time to detect, bind, and destroy leukemia cells. APVO436, in combination with Ven and Aza, achieved positive results in a phase Ib dose-escalation trial for patients with AML [157, 158]. The results revealed that the remission rate of patients receiving this triple therapy was 82% (9/11), and 27% (3/11) of patients experienced sufficient remission and could continue to receive stem cell transplantation. In addition, among the patients who achieved remission, one patient maintained complete remission for 8 cycles (the longest cycle allowed by the protocol), which means that the duration of remission (DOR) was at least 8 months. At present, the median DOR has not been reached.

JNJ-67571244 is a novel bispecific antibody developed by Johnson & Johnson that targets the conserved structural domain IgC2 of the CD33 antigen [159] and is a CD33×CD3 bispecific antibody originating from Genmab. It exhibits potent T-cell redirecting activity against AML. Research indicates that JNJ-67571244 binds to the C2 domain of CD33, demonstrating broad activity regardless of the genotype of the single-nucleotide polymorphism (SNP) rs12459419. Currently, JNJ-67571244 has completed phase I clinical trials (NCT03915379), which involve subjects with R/R AML and high-risk MDS. Although some patients experience temporary disease burden reductions, no responses are observed. JNJ-67571244 administration increased the levels of multiple cytokines, which coincided with the incidence of cytokine release syndrome (CRS), infusion‐related reactions, and elevated liver function. A prolonged step‐up strategy was tested to improve tolerability, although this approach did not prevent hepatotoxicity. T‐cell activation following treatment suggested target engagement but did not correlate with clinical activity. Safely reaching the projected exposure level for JNJ‐67571244 efficacy was not achieved; thus, the MTD and RP2D were not determined [160].

Flotetuzumab (MGD006) is an investigational bispecific antibody-based molecule for CD3ε and CD123 engineered in a DART format [161]. CD3-engaging bispecific molecules bind both tumor and effector cells to promote an immunologic synapse and redirect polyclonal effectors to kill tumor cells in a major histocompatibility complex (MHC)-independent fashion. In preclinical models, flotetuzumab mediated target‒effector cell association, T-cell activation and proliferation, and potently killed CD123^+^ AML blasts both in vitro and in vivo [162, 163]. A multicenter, open-label, phase I/II study of flotetuzumab in patients with R/R AML (NCT02152956) revealed its clinical benefit [164].

Other bispecific antibodies undergoing clinical research include drugs targeting CD33, FLT3 and WT1. Although some side effects (severe CRS or neurological toxicity) have been observed with these drugs, this treatment approach still shows promising clinical application prospects.

### Tri-specific killer engager (TriKE)

TriKE^®^ therapeutic drugs are targeted immunotherapy drugs modified by a bispecific antibody platform. The TriKE formulation is an immune cell conjugate based on camel nanoparticles designed to connect NK cells and cancer cells for the selective targeting and killing of cancer cells. The first-generation TriKE therapeutic drug consists of three functional components: a camel nanobody that binds to CD16 receptors on NK cells, a single-chain variable fragment (scFv) that recognizes markers expressed on tumor cells, and a human IL15 crosslinker. The IL-15 component of TriKE provides a self-sustaining signal that activates NK cells and enhances their ability to kill tumor cells.

GTB-3550 (OXS-3550) [165] is a single-chain three-specific single-chain recombinant fusion protein conjugate composed of variable regions of heavy and light chains of anti-CD16 and anti-CD33 antibodies and human IL-15. It enhances natural killer cell function by targeting CD33-positive malignant cells and CD33^+^ MDSCs, inducing antitumor responses that target CD33. In the completed phase I clinical study (NCT03214666), GTB-3550 was demonstrated to be safe and well tolerated at the evaluated dose. The clinical trial for GTB-3550 has currently been terminated, primarily because GT Biopharma has decided to shift its focus to the development of the second-generation TriKE product, GTB-3650. The study of the GTB-3550 demonstrated clinical proof of concept and provided a framework for future clinical trial design of TriKE candidate products. TriKEs targeting other antigens are also being studied, with both CD16-IL15-CLEC12A TriKE [166] and NKG2C-IL-15-CD33 [167] demonstrating effective in vitro killing of AML cells.

### Chimeric antigen receptor T-cell therapy (CAR-T)

CAR-T cells are modified autologous peripheral T cells that express synthesized target antigen receptors, bind to target antigens, initiate intracellular signaling, activate non-TCR-dependent T cells, target antigen-dependent amplification, and form long-term leukemia memory [168]. The CAR consists of four main components that can affect CAR-T-cell function: an extracellular target antigen-binding domain, a hinge domain, a transmembrane domain, and one or more intracellular signal domains.

The extracellular domain is important not only for antigen recognition but also for binding affinity and specificity. Low or high affinity may induce activation-induced cell death (AICD) and toxicity in CAR-T cells. The adaptability of the hinge region allows the antigen-binding domain to enter the target epitope and form immune synapses, with the most commonly used hinge regions derived from CD8, CD28, IgG1, and IgG4. The transmembrane domain affects the expression level and stability of the CAR, and most transmembrane domains originate from natural proteins, including CD3 ζ, CD4, CD8 α, and CD28. The CD3 ζ transmembrane domain can promote T-cell activation, mediate CAR dimerization, and bind to endogenous TCRs, but it is not as stable as the CD28 ζ domain. The hinge domain and transmembrane domain affect the production of cytokines and AICD in CAR-T cells. The intracellular domain affects the degree and persistence of CAR-T-cell activation. The challenge of CAR-T-cell therapy is the highly inhibited microenvironment and the expression of target antigens in normal hematopoietic cells [168, 169]. Currently, over 20 studies are underway that target antigens, including CD33, CD123, CLL1, CD38, NKG2D, and CD7.

CD123 CAR-T cells have shown strong antileukemic activity in vitro, but their application is limited because of the difficulty in distinguishing leukemia cells from normal hematopoietic cells. CD44v6 is an adhesive protein that facilitates the development of leukemia and contributes to the phenotype of LSCs. CD44v6 CAR-T cells have strong antileukemic effects in vitro and preserve normal HSCs [170, 171]. CLL1 is also a target antigen for CAR-T cells, and in vitro studies and mouse models have shown encouraging results in distinguishing normal and leukemia cells. Phase I studies of CD33-CLL1 dual CAR-T cells have been initiated, and preliminary results show that 7 out of 9 cases are MRD negative. Cui [172] studied the safety of CD38 CAR-T-cell therapy for relapsed AML patients after allo-HSCT. After 4 weeks of infusion, 4 out of the 6 patients achieved CR/CRi, and toxicity was controllable, with leukemia-free survival (LFS) lasting 7.9 months. A phase I dose study of CD33 and IL-15 CAR-T-cell therapy for R/R AML patients and high-risk MDS patients revealed an ORR of 50%, with good safety and tolerability. Preclinical studies have used dual targeted therapy with FLT3 CAR-T cells and the FLT3 inhibitor gilteritinib for the treatment of *FLT3-*mutant AML, which can effectively clear leukemia cells and preserve normal HSCs. Appelbaum used controlled CD33 CAR-T cells (SC-DARIC33) to treat R/R CD33^+^AML, which can be controlled and adjusted by intermittent use of rapamycin after infusion [173].

This prospective phase II trial (NCT03896854) evaluated the safety and efficacy of CD19 CAR-T-cell therapy in 10 patients who relapsed with CD19-positive t (8;21) AML. This study enrolled eight patients with hematologic AML and two with molecular relapsed AML. The median bone marrow blast percentage was 12.4% (0.1–50.2%), and the blasts exhibited a median CD19 positivity of 55.7% (22.6–97.1%). Genetic profiling revealed TP53 alterations (*n* = 1) and KIT (*n* = 3) and *FLT3-ITD* (*n* = 1) mutations. After lymphodepletion with fludarabine and cyclophosphamide (FC), 5–20 × 10^6^ cells per kilogram of CAR-T cells were administered. All patients experienced grade 3 or higher hematologic toxicities following tumor-reduction chemotherapy and the FC regimen, which were managed for a median of two weeks after CAR-T-cell therapy. Nonhematological toxicities were mild and reversible. Eight patients presented with mild (grade 1–2) CRS, and one experienced grade 3 CRS. Immune effector cell-associated neurotoxicity syndrome was not observed. All patients achieved CR after CAR-T-cell therapy, with 60% achieving a molecularly MRD-negative CR. RUNX::RUNX1T1 fusion transcript levels demonstrated a median 2.5-log reduction (range: 0.7–4.5 log; *P* = 0.002). At a median follow-up of 64.6 months (range: 11.2–88.8 months), the median overall survival and leukemia-free survival were 11.6 and 3.8 months, respectively. The 12-month cumulative incidence of relapse was 53.3%. These findings indicate that CD19 CAR-T cells are a safe and effective option for patients with relapsed CD19-positive t (8;21) AML [174].

PRGN-3006 was developed via Precigen’s revolutionary Ultra CAR-T-cell treatment platform, which eliminates the need for ex vivo expansion, shortens the manufacturing time, and enables the administration of CAR-T-cell therapy to patients at cancer centers just one day after nonviral gene transfer. The use of Precigen’s advanced nonviral gene delivery system to coexpress a chimeric antigen receptor, membrane-bound interleukin-15 (mbIL15), and a kill switch offers improved precision and control in targeting R/RAML and high-risk MDS. Dose escalation data for PRGN-3006 (NCT03927261) revealed robust dose-dependent expansion and persistence of PRGN-3006 in peripheral blood and bone marrow following a single infusion with no DLTs reported to date, leading to an ORR of 27% in heavily pretreated patients in the lymphodepletion cohort, which is significant for the AML patient population with limited treatment options [175].

CAR-T cells have the potential to revolutionize the treatment of hematological malignancies, but many obstacles remain to be overcome. For AML, the ideal LSC target antigen should be more specific, should be consistently expressed in all disease stages, and should be easy to monitor. CAR-T-cell production needs improvement to increase target antigen recognition, ensure sufficient binding strength to activate T cells without causing AICD, limit target toxicity, and prolong CAR-T-cell persistence [176].

### NK cell-based immunotherapies

NK cell-based therapy may provide a new therapeutic perspective and safer alternative for targeting AML cells. In addition to CAR-T-cell therapy, CAR-NK cells targeting different AML cell antigens (CD70, CD33, FLT3, and CLL1) have shown strong antileukemic effects in vitro, with strong cytotoxicity but limited off-target toxicity and unique recognition mechanisms, without the common complications of T-cell therapy, such as CRS or neurotoxicity [177, 178]. Currently, they are in the preclinical evaluation stage. CAR-NK cells show antileukemic activity against AML that is impaired by the interaction between HLA-E and the inhibitory receptor NKG2A. Bexte [179] generated CD33 specific, AML-targeted CAR-NK cells (CAR33) combined with CRISPR/Cas9-based gene disruption of the NKG2A-encoding KLRC1 gene, which overcomes CAR-NK cell inhibition mediated by the HLA-E-NKG2A immune checkpoint. CAR33-KLRC1^ko^-NK cells are preserved following exposure to AML cells. Moreover, CAR33-KLRC1^ko^-NK cells demonstrate potent antileukemic killing activity against AML cell lines and primary blasts both in vitro and in vivo. Therefore, CAR-NK cells lacking NKG2A may be able to bypass the immune suppression of AML.

Owing to the synergistic effect of the different activation receptors required for the complete activation of NK cells, antibody-based NK cell adapter (NKCE) technology to generate three functional molecules (NKp46-CD16a-NKCEs) that target antigens expressed on cancer cells has been developed and synergistically binds NKp46 and CD16a on NK cells [180]. NKp46 (NCR1, CD335) is a highly conserved activated cell surface glycoprotein in mammals. NKp46 is expressed on all NK cells, ILC1s, and very small subsets of T cells and ILC3s. The NKp46 signal mediates NK cell activation, cytotoxicity, and cytokine release through its association with CD3 ζ and FcR γ. SAR443579 (SAR’579) [181] is a triple functional anti-CD123-NKp46-CD16 NKCE. At a dose of 3000 µg/kg once a week, SAR’579 monotherapy showed good tolerability, and no dose-limiting toxicity was observed. The most common TRAE was an infusion-related reaction, which occurred in 13 (56.5%) patients. When a maximum target dose of ≥ 1000 µg/kg was used, 3/16 (18.8%) patients achieved CR/CRi. Two patients who achieved remission still received maintenance treatment at the end of the follow-up period.

Recently, cell-based immunotherapy using NK cells has attracted considerable attention in the context of cancer immunotherapy. NK cells generated from induced pluripotent stem cells (iPSCs) constitute a new option for use as an NK cell resource. CD226 plays a vital role in NK cell cytotoxicity, interacting with its ligands on tumor targets. AML cells have developed mechanisms to escape NK cell cytotoxicity, including the downregulation of CD226 on NK cells. Induced pluripotent stem cell-derived NK (iPSC-NK) cells constitute an important source of standardized off-the-shelf NK cell therapy for treating AML patients. iPSC-derived NK cells engineered with CD226 represent promising candidates for off-the-shelf immunotherapy, particularly in AML and other CD226 ligand-expressing malignancies [182].

### Peptide vaccines

Peptide vaccines are generally selected from a segment of malignant tumor antigens and are structurally modified by means such as amino acid substitution [183]. The modified peptides have stronger immunogenicity and can stimulate the immune response of patients after subcutaneous inoculation. The immune system of patients can recognize and eliminate cancer cells by identifying the specific antigens of the cancer cells. Currently, various peptide vaccines targeting leukemia-related antigens, including the WT1 antigen, PR1, PR3, PRAME, and RHAMM, which are the most common vaccine targets, have been developed [184–186]. Peptide vaccines have disadvantages such as poor immunogenicity, easy metabolism and digestion by the body, and induction of immune tolerance. The search for ideal adjuvants in the research of peptide vaccines to increase the immunogenicity of vaccines is highly important. The *WT1* gene is abnormally highly expressed in AML, is restricted to normal tissues, and is related to the poor prognosis of AML patients. Therefore, it is considered an AML antigen and can be used as a new target for specific immunotherapies [184, 187–189]. A phase II clinical trial (NCT01266083) revealed that the median PFS of patients in first clinical remission who were treated with the WT1 vaccine, galinpepimut-S, was 16.9 months, and the vaccine was well tolerated [190]. Additionally, phase III studies are currently being conducted [191]. The combined treatment strategy of CAR-T-cell therapy and vaccines has made progress in various solid tumors and malignant hematological tumors. Animal experiments have shown that tumor-specific antigens (such as WT1) can increase the expansion and activation of specific CAR-T cells in mouse models after CAR-T-cell inoculation. The addition of immune stimulants, including adjuvants, co-stimulators and cytokines, helps improve the efficacy of vaccines, especially.

## Future directions in AML

**Fig. 2:**
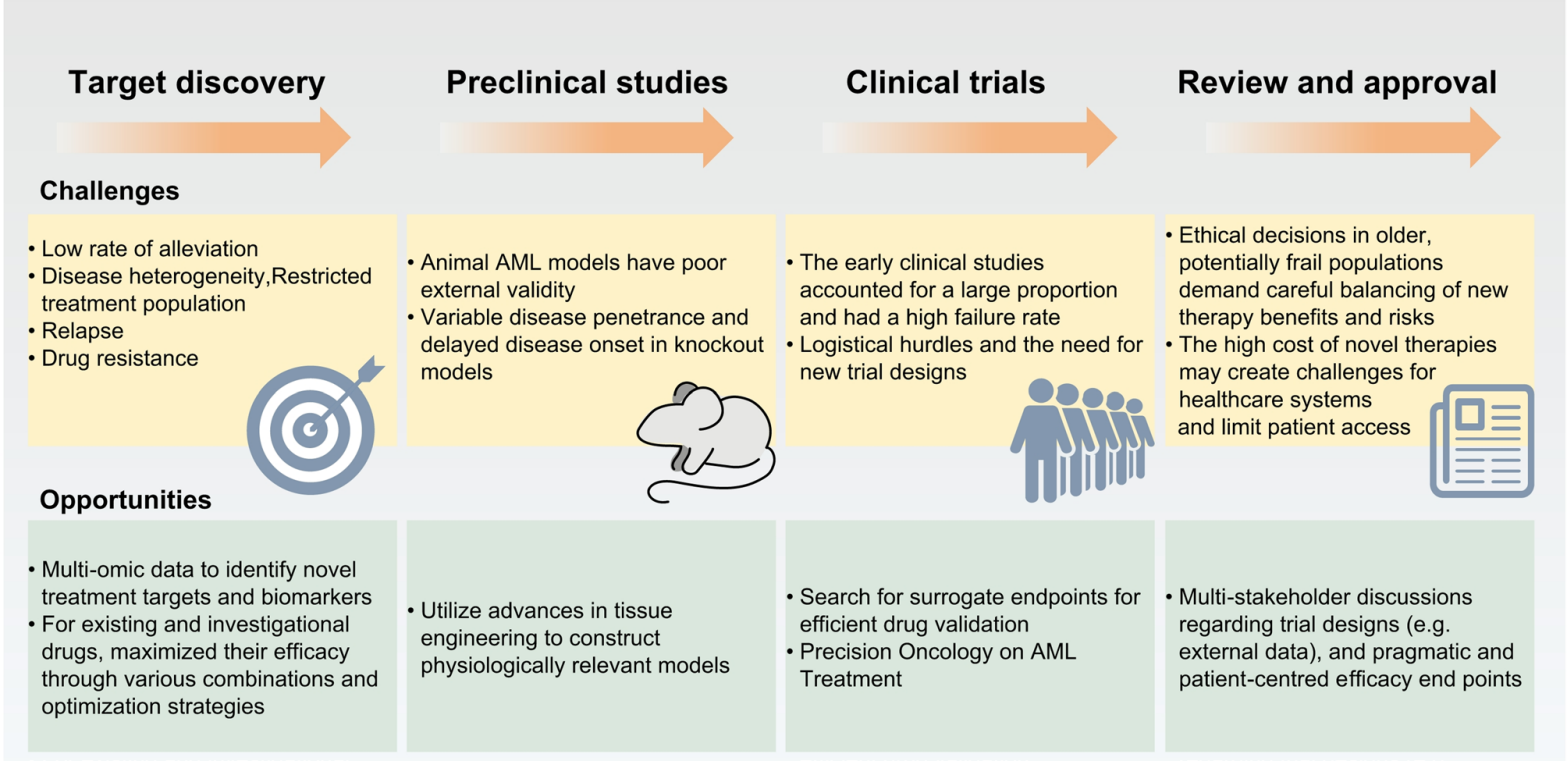
Challenges and opportunities for AML drug development. The development of AML therapeutics faces major challenges, including incomplete understanding of pathogenesis, a scarcity of predictive biomarkers, and high clinical trial attrition rates. Key opportunities for optimization exist across all stages of drug discovery: machine and deep learning can analyze multiomics data from well-phenotyped patients to uncover novel targets and biomarkers; these can be validated using physiologically relevant organoid models to improve preclinical predictability. Furthermore, rationally designed clinical trials may significantly enhance translation and success rates

With advancements in medical research, new drugs and treatment strategies continue to emerge, providing more possibilities for the treatment of AML. During the treatment process, doctors tailor the best treatment plan on the basis of the latest research findings and personalized assessments. AML highlights strengths in ongoing research and evolving treatment options, but weaknesses include significant side effects and limited biomarkers. Opportunities lie in improving therapies and identifying reliable biomarkers, whereas threats involve resistance to new agents, high treatment costs, and ethical considerations in older populations. Addressing weaknesses and leveraging strengths offers opportunities to overcome threats and improve AML treatment outcomes. The future landscape is likely to incorporate AI-driven treatment algorithms that integrate multiomics data, patient-derived organoid models for drug sensitivity testing, and novel clinical trial designs (such as umbrella and basket trials) to accelerate the development of personalized therapies (Fig. 2).

Several key future trends in AML therapy are described below.

### Personalized immunotherapy

Future AML treatments will increasingly rely on patient-specific characteristics, including genetic mutation profiles, epigenetic changes, and the state of the immune microenvironment. Through high-throughput sequencing and multiomics analysis, doctors can tailor the most appropriate immunotherapy regimen for each patient. Bradley [192] annotated cells with detected mutations that were classified along this axis as HSC-like, progenitor-like, GMP-like, promonocyte-like, monocyte-like or cDC-like malignant cells, and differentiated monocyte-like AML cells expressed diverse immunomodulatory genes and suppressed T-cell activity in vitro.

### Optimization and expansion of CAR-T cell therapy

#### Multitarget CAR-T cell

While current CAR-T-cell therapies primarily target CD19 in lymphoid leukemias, researchers are exploring multitarget CAR-T cells for AML because of the lack of ideal single targets. These cells can simultaneously target multiple AML-associated antigens, reducing off-target effects and resistance [193].

#### Enhancing CAR-T-cell persistence and function

Gene editing technologies (such as CRISPR/Cas9) or the introduction of costimulatory molecules can increase the persistence and antitumor activity of CAR-T cells, particularly in the complex immunosuppressive microenvironment of AML [194, 195].

#### Novel CAR structures

Developing CARs with greater selectivity and lower toxicity, such as logic-gated CARs that activate T cells only when multiple signals are present, can improve safety [196, 197].

### Development of TCR-T cell therapy

#### Targeting intracellular antigens

In contrast to CAR-T cells, TCR-T cells can recognize intracellular antigen peptides, offering a unique advantage in AML treatment. Future research will focus on identifying more AML-related intracellular antigens and developing efficient TCR-T cells that target these antigens [198–200].

#### Overcoming immune evasion

Modifying TCR-T cells to better overcome immune evasion mechanisms in AML, such as by blocking PD-1/PD-L1 pathways or other immune checkpoints, can increase their effectiveness [201].

### Modulation of the tumor microenvironment

#### Reversing immunosuppressive microenvironments

The AML tumor microenvironment contains various immunosuppressive factors, such as Tregs, MDSCs, and immunosuppressive cytokines (such as IL-10 and TGF-β) [192, 202]. Future research will aim to develop strategies to reverse these inhibitory factors, such as the use of small molecule inhibitors or antibodies to deplete Tregs and MDSCs or block immunosuppressive cytokines.

#### Promoting antitumor immune responses

The activation of dendritic cells (DCs) or NK cells plays a crucial role in enhancing antitumor immune responses. For instance, Toll-like receptor (TLR) agonists or GM-CSF can effectively promote DC maturation, thereby potentiating antigen presentation and strengthening T-cell-mediated immunity against tumors [203].

### Exploration of emerging immunotherapies

#### Macrophage reprogramming

The AML tumor microenvironment is rich in M2-type macrophages, which typically have immunosuppressive functions. Reprogramming these macrophages to shift them from the M2 phenotype to the M1 phenotype (which has antitumor activity) can increase the effectiveness of immunotherapy [204].

### Application of artificial intelligence

Leveraging machine learning and artificial intelligence to analyze large datasets of clinical, genomic, and immunomic data can help predict patient responses to different immunotherapies and guide personalized treatment decisions. AI can assist in designing optimal combination therapies and dosing schedules, improving treatment outcomes and minimizing side effects [205].

Future therapy for AML is moving toward a more personalized, precise, and integrative approach. With the emergence of new technologies and the advancement of clinical trials, immunotherapy has become a core component of AML treatment, significantly improving patient survival and quality of life. However, the complexity and heterogeneity of AML still present many challenges, necessitating interdisciplinary collaboration and continuous innovative research to further advance this field. For example, some younger/fit AML subsets are highly resistant to intensive chemotherapy. These include *TP53*-mutated AML, *MECOM*‐AML, and secondary treated AML. Such patients should ideally be referred immediately for investigational approaches and analyzed separately to dissect more precisely the benefit of novel strategies in such very high‐risk AML (expected CR rates from < 40% to 50%; historical 12‐month survival rates, < 20%) vs. other AMLs [3]. Moreover, drug development needs to balance effectiveness and safety, and toxicity is a challenge. The influence of the occurrence and severity of AEs still needs to be confirmed in more clinical trials and in practice. For example, the CD3 BiTE commonly has a high incidence of CRS, and the CD123/CD3 candidate drug XmAb14045 (vibecotamab) of Novartis has serious safety issues in clinical trials. One patient developed CR after the first dose, and another patient developed acute pulmonary edema after multiple doses, ultimately leading to the termination of product development. The clinical trial of JNJ63709178, a CD123/CD3 bispecific antibody developed in collaboration between Johnson & Johnson and Genmab, has been completely halted by the FDA because of severe toxic side effects. Compared with decitabine alone, talacotuzumab, another monoclonal antibody of Johnson and Johnson that targets CD123, was found to be associated with a higher rate of serious treatment-related AEsin combination with decitabine. Owing to the lack of efficacy and high toxicity rate, Johnson and Johnson ultimately decided to discontinue the entire talacotuzumab study program.

## Conclusion

Advances in molecular profiling technologies have substantially deepened our understanding of the genetic and epigenetic alterations underlying AML, unveiling a range of novel therapeutic targets. As a result, targeted molecular agents and epigenetic therapies are playing an increasingly vital role in treatment strategies. Additionally, immunotherapy has emerged as a highly promising approach, demonstrating significant potential in AML management. To address ongoing challenges—such as the identification of suitable targets, management of treatment-related toxicity, and mitigation of immune-related adverse effects—further research is essential to translate these advances into clinical benefits and meet unmet medical needs.

## Data Availability

No datasets were generated or analysed during the current study.

## Abbreviations

AML: Acute myeloid leukemia
AICD: Activation-induced cell death
AEs: Adverse events
allo-HSCT: Allogeneic hematopoietic stem cell transplantation
ADCC: Antibody-dependent cellular cytotoxicity
ADCP: Antibody-dependent cellular phagocytosis
APCs: Antigen-presenting cells
Aza: Azacitidine
BRD4: Bromodomain-containing protein 4
CIBMTR: Center for international blood and marrow transplant research
CDC: Complement-dependent cytotoxicity
CR: Complete remission
CRi: Complete remission with incomplete count recovery
CRS: Cytokine release syndrome
CTL: Cytotoxic T lymphocyte
Dec: Decitabine
DARPin: Designer ankyrin repeat protein
DOT1L: Disruptor of telomeric silencing 1-like
DNMTs: DNA methyltransferases
DLI: Donor lymphocyte infusion
DOR: Duration of remission
EZH2: Enhancer of zeste homolog 2
ELN: European leukemia net
EFS: Event-free survival
FOs: Fusion oncoproteins
GvHD: Graft-versus-host disease
GvL: Graft-versus-leukemia
HSCs: Hematopoietic stem cells
HDACs: Histone deacetylases
HL: Hodgkin's lymphoma
HMAs: Hypomethylating agents
IED: Immune effector dysfunction
IV: Intravenous
Lena: Lenalidomide
LFS: Leukemia-free survival
LSCs: Leukemic stem cells
KAT6A: Lysine acetyltransferase 6A
MTD: Maximum tolerated dose
MRD: Measurable residual disease
mOS: Median OS
MDS: Myelodysplastic syndromes
MDSCs: Myeloid-derived suppressor cells
MPN: Myeloproliferative neoplasms
NK cells: Natural killer cells
NHL: Non-hodgkin lymphoma
NRM: Non-relapse mortality
ORR: Objective response rate
OS: Overall survival
PNH: Paroxysmal nocturnal hemoglobinuria
PTM: Posttranslational modification
RP2D: Recommended phase II dose
RFS: Recurrence free survival
R/R: Refractory/relapsed
Treg: Regulatory T
SIRP α: Signal regulatory protein alpha
SNP: Single-nucleotide polymorphism
SUMOylation: Small ubiquitin-related modifier
TCR: T cell receptor
TAGs: Targeted antibody‒drug conjugates
TET: Ten-eleven translocation
TOP1i: Topoisomerase 1 inhibitor
VEN: Venetoclax
